# Remission induction therapies and long-term outcomes in granulomatosis with polyangiitis and microscopic polyangiitis: real-world data from a European cohort

**DOI:** 10.1007/s00296-024-05757-4

**Published:** 2024-12-24

**Authors:** Stefan Krämer, Kristian Vogt, Theresa Maria Schreibing, Martin Busch, Tobias Schmitt, Raoul Bergner, Sebastian Mosberger, Thomas Neumann, Thomas Rauen

**Affiliations:** 1 Department of Nephrology and Clinical Immunology, RWTH Aachen University Hospital, Pauwelsstraße 30, 52074 Aachen, Germany; 2 Department of Internal Medicine III, University Hospital Jena, Friedrich-Schiller University, Jena, Germany; 3 Department of Internal Medicine A, Nephrology and Rheumatology, Municipal Hospital Ludwigshafen, Ludwigshafen, Germany; 4 Department of Rheumatology and Immunology, Cantonal Hospital St. Gallen, St. Gallen, Switzerland

**Keywords:** Vasculitis, AAV, ANCA, MPA, GPA, Relapse, Rituximab, Cyclophosphamide, Renal outcome

## Abstract

To explore disease characteristics, renal involvement and induction treatment strategies over the last decades and evaluate relapse rates and renal outcomes in ANCA-associated vasculitides (AAV). We retrospectively analyzed remission, relapse rates and the occurrence of the composite endpoint (comprising death and renal failure) in newly diagnosed AAV cases in four tertial referral centers in Germany and Switzerland diagnosed between 1999 and 2022. Hazard ratios were computed by Cox proportional hazard and Kaplan–Meier curves were plotted to compare therapeutic strategies after propensity-matching. In our cohort of 358 AAV patients, 203 (58.1%) were classified as granulomatosis with polyangiitis (GPA) based on the novel 2022 ACR/EULAR criteria, 139 (38.8%) as microscopic polyangiitis (MPA). The proportion of MPA cases among all AAV patients increased from 28.9% between 1999 and 2013 up to 46.7% thereafter. Cyclophosphamide (CYC) was chosen most frequently for remission induction (74.8% before 2013 and 57.3% thereafter), whereas the use of rituximab (RTX) increased from 5 to 26% within these periods. GPA patients had a higher relapse rate as compared to MPA patients (41.3% vs. 25.9%, p = 0.006). However, in AAV patients with renal involvement, renal events (i.e. end-stage kidney disease or a persistent drop in the estimated glomerular filtration rate (eGFR) below 15 ml/min/1.73 m^2^) occurred more frequently in MPA patients, particularly under RTX treatment as compared to matched CYC counterparts (11.8% vs. 7.5%, p = 0.011). In our cohort, GPA patients exhibited frequent relapses, advocating for a more intense and extended treatment. MPA patients had lower relapse rates, however, RTX was less effective to prevent renal endpoints in MPA as compared to CYC, highlighting the need to further investigate additional treatment strategies.

**Supplementary Information:**

The online version contains supplementary material available at 10.1007/s00296-024-05757-4.

## Introduction

In the last decade, the therapeutic armamentarium for patients with anti-neutrophil cytoplasmatic antibody (ANCA)-associated vasculitides (AAV) has been significantly enriched by the B-cell-depleting antibody rituximab (RTX) which demonstrated non-inferiority over cyclophosphamide (CYC) in induction of remission [1, 2]. RTX has been approved by the European Medicine Agency (EMA) for AAV in 2013. Still, renal involvement remains a major burden of disease, demanding renal replacement therapy in severe cases. Long-term follow-up showed better outcomes with reduced mortality and relapse rates and lower occurrence of end-stage kidney disease (ESKD) in AAV patients with renal involvement receiving RTX in addition to low-dose pulsed CYC for induction as compared to propensity-matched AAV cases from the European Vasculitis Society (EUVAS) [3]. In 2022, ACR and EULAR proposed novel classification criteria for granulomatosis with polyangiitis (GPA) [4] and microscopic polyangiitis (MPA) [5] highlighting the distinguished importance of enzyme-linked immunosorbent assay (ELISA) readings for the classification of AAV subsets. As such, the proof of antibodies against proteinase-3 (PR3) adds five points to GPA [4] and that of anti-myeloperoxidase (MPO) antibodies adds six points to the MPA criteria [5], representing the highest score values within these classification criteria, respectively. Recent data indicate significant genetic differences accompanied with both entities [6] which could yet not be translated into clinical presentation or choice of therapeutic strategies. Recently, we described distinct patterns of pulmonary affection in computed tomography with regards to clinical outcomes [7]. Therefore, exploring divergent responses based on GPA vs. MPA classification to current therapeutic regimens appears to be a key research question. Globally, the distribution between GPA and MPA varies among AAV patients between countries: GPA was found to be predominant among AAV patients in North Europe, Middle East, Turkey, and India, whereas MPA was found more prevalent in Asian AAV cohorts [8].

In the present analysis, we retrospectively collected data from a large multicentric cohort of European AAV patients that were diagnosed and treated between 1999 and 2022. We aimed to characterize baseline characteristics, clinical courses and treatment responses including remission and relapse rates. Since our cohort comprised a majority with renal vasculitis involvement, we focused on mortality and renal outcomes in this particular subcohort.

## Methods

### Study population

We included newly diagnosed AAV cases from three German (Aachen, Jena, Ludwigshafen) and one Swiss (St. Gallen) tertiary rheumatology and/or nephrology referral centers between 1999 and 2022 with available follow-up data of at least one year upon diagnosis of AAV. Re-classification of the disease according to the new 2022 ACR/EULAR criteria was performed algorithmically as previously described [9]. We retrospectively analyzed baseline parameters including organ involvement, Birmingham Vasculitis Activity Score (BVAS, Version 3), laboratory, and histological findings at the time of diagnosis, therapeutic agents for induction and outcome as well as organ damage measures. Data were collected from records of routine clinical visits also including hospitalizations as deposited in the medical information systems at each center.

### Influence factors and medications

We conducted an analysis of biometric data, focusing on relevant baseline patient characteristics, including concomitant disease conditions and therapeutic approaches, particularly induction therapies including CYC or RTX based on established protocols. CYC and RTX were considered primary remission induction therapies and were analyzed separately from patients who received methotrexate (MTX), azathioprine (AZA), mycophenolate mofetil (MMF) as primary initial agents or were treated with corticosteroid monotherapy. Therapeutic regimen comprising either RTX or CYC within 90 days after prior treatment with the relative counterpart were considered combination therapy, regardless of whether it was intended to enhance immunosuppression or due to intolerance or toxicity. Maintenance therapy was defined as the drug administered continuously for at least three months following remission induction (CYC, RTX, or combination). Plasmapheresis (PLEX) was considered as additional treatment. Corticosteroid therapy was initiated as specified in the current guidelines at the time, either as intravenous bolus therapy followed by oral treatment and tapering or started orally with subsequently tapering to a target dose of 5 mg/day over three to six months. This lower dosage was maintained for varying durations.

### Outcome measures

Patient outcomes were recorded after six and twelve months by scheduled routine visits. Additional follow-up visits were documented in case of a relapse as captured during visits or hospitalization in the following years. If no such event was documented, last patient contact was used as the end of follow-up entry. Death and cause of death were captured. Remission status was reported after twelve months whereas complete remission (CR) was defined as a BVAS of 0. A relapse was defined as any vasculitis-related disease activity, assessed by BVAS ≥ 1, necessitating therapy intensification beyond a daily glucocorticoid dosage of 10 mg prednisolone, or if relapse was reported in any patient record. The composite renal endpoint encompassed all-cause death and renal events such as ESKD (i.e. necessity for renal replacement therapy or a persistent drop in the estimated glomerular filtration rate (eGFR) below 15 ml/min/1.73 m^2^ at six months after AAV diagnosis or later [10]).

### Statistics

For time-series analysis, the data set was divided into two-time intervals: patients that were diagnosed and treated between 1999 and 2013 and those that were diagnosed afterwards. The year 2013 was selected as the watershed because RTX was licensed for AAV and available in daily use in many European countries around this time, and 2013 is close to the median of the entire data collection period.

GFR was estimated using the 2009 Chronic Kidney Disease Epidemiology Collaboration (CKD-EPI) formula. Statistical analyzes were performed by ANOVA for metric values and Fisher’s exact/Chi^2^ test for frequencies, metric means are given ± standard deviations (SD), median values in addition to the interquartile ranges (IQR). To obtain a broad overview of possible influencing factors for relapse or renal events, odds ratios were computed applying Cox proportional hazard regression and visualized as forest plots ± 95% confidence interval for the whole study population and distinct subsets. Afterwards, Kaplan–Meier curves were plotted for event analyzes in all cases and after propensity-matched renal cases for age, sex, body mass index (BMI), baseline C-reactive protein (CRP) and eGFR for comparison between GPA and MPA as well as between CYC- and RTX-induced patients. P-values below 0.05 were regarded significant. Computations and plotting were performed using Python 3, R software, version 3.3.1 (R Foundation for Statistical Computing) using ‘clinicopath’ and ‘clinicopathsurvival’ (0.0.2.0093, by Serda Baldaci), propensity matching was performed by ‘MatchIt’ (3.0.2) library [11] for R software packages.

## Results

### Patient characteristics

Since 1999 a total of 358 cases were identified with available follow-up visit entries exhibiting a median age of 61 years (IQR 50–70 years) and more than 95% of Caucasian descent, 168 (46.9%) were females (Table 1). Across all cases, the mean observation time was 5.8 years within a range of 131 days up to twenty-one years. According to the novel 2022 ACR/EULAR criteria, we identified 208 individuals (58.1%) as GPA and 139 (38.8%) as MPA [4, 5]. Eleven cases (3.1%) could not be classified based on these criteria (Table 1). Three of the four Asian patients had diagnosis of an MPA. Following re-classification, we observed an increase in MPA cases compared to the initial clinical diagnosis. This change is primarily due to 29 cases, initially diagnosed as “renal limited AAV”, being re-classified as MPA and only three as GPA (suppl. table 1). We calculated high concordance rates (90% for GPA an 98% for MPA patients) between initial diagnoses and those based on the novel criteria as previously demonstrated [9]. Nine of the eleven “unclassified cases” did not display PR3- or MPO-specificity at the time of initial diagnosis, two cases were PR3-positive. “Unclassified patients” showed predominantly ENT (7/11), pulmonal (6/11), and in minority renal involvement (4/11). The evaluation of relevant concomitant conditions revealed no significant differences between both groups in smoking, hypertension, or coronary artery disease except for preexisting renal disease in the MPA group. Overall, MPA patients were significantly older at diagnosis (65.0 vs 58.2 years), whereas gender distribution was almost balanced between both groups. At initial presentation, GPA patients exhibited higher disease activity in general as expressed by increased BVAS scores than MPA patients (16.2 vs.13.7). In more detail, renal AAV affection was found more frequent in the MPA group (84.9 vs. 68.3%) and was significantly more severe as documented by lower baseline eGFR (37.3 vs. 59.2 ml/min/1.73 m^2^ in GPA patients). Organ involvement was more heterogeneous among GPA patients with more prominent general, ENT, lung, neurological and mucosal manifestations.

**Table 1:** Baseline characteristics, comorbidities, organ manifestations, vasculitis associated activity and outcome

		ACR/EULAR 2022 classification			*p*
	Overall	GPA	MPA	Not class	GPA vs. MPA
*N*	358	208	139	11	
Baseline characteristics					
Female Sex, *n* (%)	168 (46.9)	98 (47.1)	65 (46.8)	5 (45.5)	0.993
Age, median [Q1, Q3]	61.0 [50.0,70.0]	58.5 [48.0,67.0]	65.0 [56.0,75.0]	61.0 [48.5,68.0]	< 0.001
BMI, mean (SD)	26.9 (5.4)	27.0 (5.4)	26.9 (5.3)	26.5 (6.5)	0.865
Caucasian, *n* (%)	342 (95.5)	202 (97.1)	132 (95.0)	8 (72.7)	
Asian, *n* (%)	4 (1.1)	1 (0.5)	3 (2.2)	0	< 0.001
Other/ unknown ethnicity, *n* (%)	12 (3.4)	5 (2.4)	4 (2.9)	3 (27.3)	
eGFR baseline, mean (SD)	51.4 (36.2)	59.2 (37.7)	37.3 (28.2)	82.3 (36.9)	< 0.001
Smoking active, *n* (%)	59 (16.7)	30 (14.6)	25 (18.2)	4 (36.4)	0.373
Included after 2013, *n* (%)	204 (57.0)	102 (49.0)	95 (68.3)	7 (63.6)	0.022
BVAS, mean (SD)	15.1 (6.9)	16.2 (7.6)	13.7 (5.3)	12.2 (8.0)	< 0.001
BVAS (renal), mean (SD)	7.6 (5.0)	6.8 (5.2)	9.0 (4.4)	3.3 (4.9)	< 0.001
Comorbidities					
Diabetes, *n* (%)	38 (10.6)	22 (10.6)	15 (10.8)	1 (9.1)	0.949
Hypertension, *n* (%)	182 (50.8)	97 (46.6)	80 (57.6)	5 (45.5)	0.046
Coronary artery disease, *n* (%)	39 (10.9)	19 (9.1)	20 (14.4)	0	0.873
Chronic kidney disease, *n* (%)	37 (10.3)	14 (6.7)	23 (16.5)	0	0.007
Neurological disorder, *n* (%)	13 (3.6)	6 (2.9)	7 (5.0)	0	0.465
AAV associated manifestations					
General, *n* (%)	220 (61.5)	145 (69.7)	69 (49.6)	6 (54.5)	< 0.001
Renal, *n* (%)	264 (73.7)	142 (68.3)	118 (84.9)	4 (36.4)	< 0.001
Chest, *n* (%)	184 (51.4)	124 (59.6)	54 (38.8)	6 (54.5)	< 0.001
ENT, *n* (%)	127 (35.5)	104 (50.0)	16 (11.5)	7 (63.6)	< 0.001
Cardiovasc, *n* (%)	13 (3.6)	9 (4.3)	4 (2.9)		0.629
Neurological, *n* (%)	56 (15.6)	42 (20.2)	12 (8.6)	2 (18.2)	0.004
Mucous, *n* (%)	53 (14.8)	45 (21.6)	7 (5.0)	1 (9.1)	< 0.001
Skin, *n* (%)	39 (10.9)	29 (13.9)	10 (7.2)		0.051
Abdominal, *n* (%)	11 (3.1)	9 (4.3)	2 (1.4)		0.132
Outcomes					
CR after 1 year, *n* (%)	276 (80.9)	161 (79.7)	109 (83.8)	6 (66.7)	0.344
VDI after 1 year, mean (SD)	1.8 (1.6)	1.7 (1.5)	1.9 (1.7)	2.2 (1.5)	0.618
VDI end of follow-up, mean (SD)	2.6 (2.2)	2.5 (2.2)	2.8 (2.3)	2.9 (1.2)	0.422
eGFR after 1 year, mean (SD)	56.6 (29.6)	63.7 (29.1)	44.8 (26.9)	66.0 (25.4)	< 0.001
Composite renal endpoint, *n* (%)	91 (25.4)	44 (21.2)	44 (31.7)	3 (27.3)	0.028
Number of first relapses	124 (34.6)	86 (41.3)	36 (25.9)	2 (18.2)	0.006
Months to first relapse, median [Q1, Q3]	33.3 [13.2,58.0]	28.3 [14.1,57.4]	35.4 [8.7,58.2]	49.4 [43.4,55.4]	0.706
Relapse or death ≤ 5 y, *n* (%)	111 (31.0)	76 (36.5)	33 (23.7)	2 (18.2)	0.024

BMI: body mass index; CAD: coronary artery disease; BVAS, Birmingham Vasculitis Activity Index; ENT, ear/nose/throat; VDI, Vasculitis Damage Index; EOF, end-of-follow-up; eGFR, estimated glomerular filtration rate, CR: complete remission (BVAS = 0), Compound renal endpoint (death, dialysis, renaltransplantation, eGFR < 15 ml/min/1.72m^2^

### Proportion of GPA and MPA cases over time

**Fig. 1:**
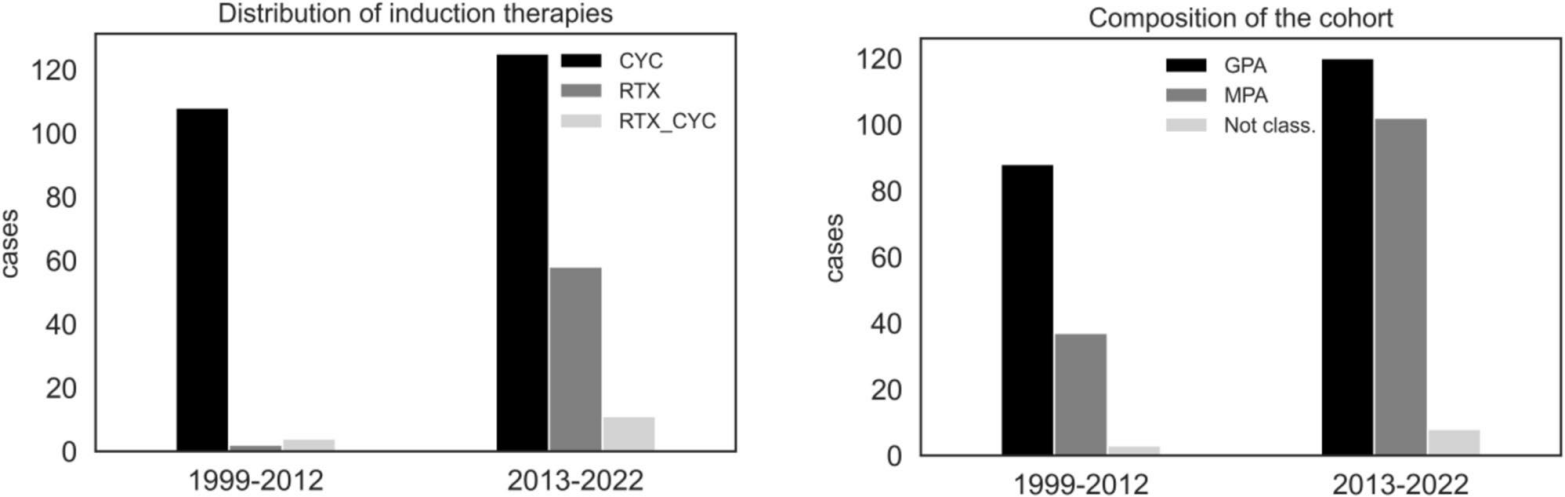
Classification and therapy for induction over time

We observed a more pronounced increase in anti-MPO^+^/MPA cases between 1999 and 2022 as compared to anti-PR3^+^/GPA cases (Fig. 1) across all four centers. When dividing all AAV cases into those diagnosed up to and from 2013 which almost represents the median follow-up period of the data collection, the proportion of MPA cases increased from 29.1% up to 44.8% among all AAV cases, whereas GPA cases decreased from 68.3% to 52%.

### Induction and maintenance therapies

Over the entire observational period, the majority of AAV patients (65%) received CYC for remission induction, whereas RTX was only used in a minor fraction (16.8%, Table 2). Fifteen patients (4.2%) received a CYC/RTX combination regimen, and 50 patients (14.0%) received none of these classic induction therapies, but were treated with methotrexate (MTX, 5%), azathioprine (AZA, 1.4%), mycophenolate mofetil (MMF, 0.3%) or corticosteroid monotherapy (3.9%). Patients that received a combined CYC/RTX induction regimen (n = 15) were around 55 years old, 67% had GPA and mean BVAS score ranged around 17, thus comparable to the main cohort. In nine AAV patients, CYC was used as initial agent followed by RTX within 90 days after diagnosis (vice versa in six patients). Overall this subgroup showed more multi-organ involvement than in the entire cohort. Upon remission induction, maintenance therapy was conducted with azathioprine (AZA) in 154 patients (43.0%) and RTX in 48 patients (13.4%). Sixty-four patients (17.9%) received low-dose corticosteroids without additional continuous immunosuppression. Only few patients were treated with MTX (12%), MMF (11.5%), or leflunomide (LEF, 2.2%) as maintenance therapy.

**Table 2:** Therapies used for remission induction and maintenance

Therapies	ACR/EULAR 2022 classification				
*N*, (%)	Overall	GPA	MPA	Not class	*p* value
Induction	358	208	139	11	
CYC	233 (65.1)	134 (64.4)	93 (66.9)	6 (54.5)	0.735
RTX	60 (16.8)	35 (16.8)	24 (17.3)	1 (9.1)	
RTX + CYC	15 (4.2)	10 (4.8)	4 (2.9)	1 (9.1)	
Other^1^	50 (14.0)	29 (13.9)	18 (12.9)	3 (27.3)	
Steroids pulses^2^	235 (65.6)	132 (63.5)	99 (71.2)	4 (36.4)	
Maintenance					
AZA	154 (43.0)	96 (46.2)	53 (38.1)	5 (45.5)	0.024
RTX	48 (13.4)	30 (14.4)	18 (12.9)		
MTX	43 (12.0)	31 (14.9)	11 (7.9)	1 (9.1)	
MMF	41 (11.5)	20 (9.6)	20 (14.4)	1 (9.1)	
LEF	8 (2.2)	1 (0.5)	7 (5.0)		
Steroids only	64 (17.9)	30 (14.4)	30 (21.6)	4 (36.4)	

^1^Other therapies than RTX, CYC or their combination for induction in all AAV-cases consist of 18 cases with MTX (5%), 5 AZA (1.4%), 1 MMF (0.3%) and 14 cases (3.9%) of steroids only, in the remaining 12 (3.4%) cases no continuously administered therapy for induction could be reconstructed

^2^Steroids either as intravenous (i.v.) boli or orally were used during induction in 92.3% (92.3% of GPA, 92.8% of MPA, and in 81.8% of not classified patients)

*CYC* cyclophosphamide, *RTX* rituximab, *AZA* azathioprine, *MTX* methotrexate, *MM* mycophenolate mofetil, *LEF* leflunomide

The most common combination regimen for induction/maintenance therapy was CYC followed by AZA (37.2%). Since RTX was first licensed in 2013 for AAV treatment by the European regulatory authorities, the majority of RTX was administered thereafter in the participating centers and was used for both, remission induction and maintenance in 20 patients (5.6% of the entire cohort). A combination of CYC for induction and RTX for maintenance was applied in 19 cases (5.3%). Virtually all patients (97.2%) received corticosteroids (i.e. up to three intravenous boli of ≥ 250 mg methylprednisolone per day followed by oral corticosteroids with subsequent tapering regimen over three months to ≤ 10 mg prednisolone per day). PLEX was performed in 75 cases (20.9%) with decreasing frequency over the entire observational period (26.4% before 2013 vs. 16.6% thereafter, p = 0.032, supplemental Table 2).

Comparing therapeutic strategies before 2013 to those within the following years, CYC was used as major remission induction therapy within both intervals, yet with a decreasing frequency from 74.8 to 57.3%. By contrast, we observed an increase in the use of RTX since 2013 for both remission induction (from 5 to 26.1%) and for maintenance (from 3.8 to 21.1%).

### Remission and relapse rates

Overall, 80.9% of patients were in complete remission one year after diagnosis irrespective of GPA or MPA cases (79.7 vs. 83.8%, Table 1). Within each classification group, the distribution of remission induction therapies was similar between GPA and MPA (Table 2). For remission maintenance, we observed minor differences with AZA, RTX, and MTX being more frequently applied in GPA patients, whereas corticosteroid monotherapy for maintenance was used more often in MPA patients.

**Fig. 2:**
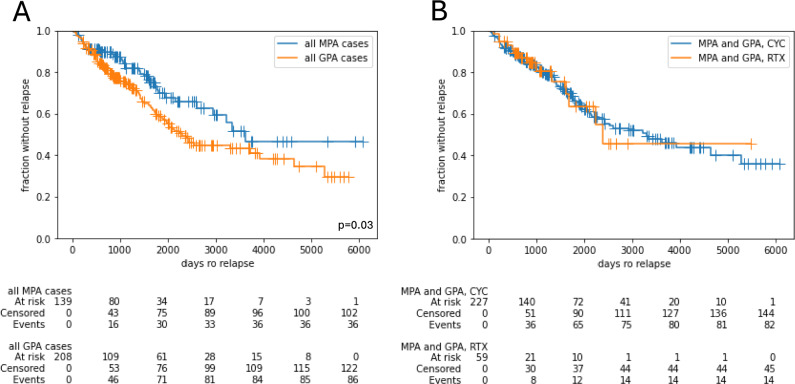
Relapse events regarding classification (**A**) and therapy for induction (**B**)

Over a median observational period of 57 months, the first relapse occurred more frequently in GPA cases (41.3% vs. 25.9% in MPA, p = 0.006). Furthermore, relapses occurred somewhat but not significant earlier in GPA patients, i.e. after a median of 28.3 months (IQR 14.1 to 57.4) vs. 35.4 months in MPA patients (IQR 8.7 to 58.2, p = 0.706) as illustrated by Kaplan–Meier plots (Fig. 2A). In the entire cohort, the chosen therapeutic regimen (CYC vs. RTX) did not impact the occurrence of relapses (Fig. 2B).

### Influence factors for relapse

We included several baseline factors that potentially might influence relapses such as vasculitis activity, inflammatory status as measured by CRP and organ involvement, in a Cox proportional hazard model. In this analysis, GPA classification emerged as the most significant factor (HR 1.58, 95% CI 1.06 to 2.36, p = 0.024, suppl. table 4). Additionally, high BVAS scores, male gender, and active smoking status were also associated with higher relapse rates. Among disease domains, the presence of general symptoms was significantly linked to an increased risk of relapse. Additionally, skin involvement and ENT manifestations, the latter serving as a surrogate for GPA, contributed to an increased risk for relapse, albeit to a lesser extent. Notably, general symptoms were present in 70% of GPA cases, while they were present in only 50% of MPA cases.

### Renal outcomes

Subsequently, we focused exclusively on patients with renal involvement according to BVAS entries (n = 264, 73.7% of the entire cohort). In this sub-cohort, renal affection was more dominant in MPA patients (84.9%) as compared to GPA cases (68.3%). Along these lines, baseline eGFR was significantly lower in MPA (37.3 ml/min/1.73 m^2^) vs. 59.2 ml/min/1.73 m^2^ in GPA (p < 0.001, Table 1). In addition, MPA patients were significantly older (65.0 years vs. 58.5 years in GPA; p < 0.001). According to induction therapy, the composite renal endpoint occurred in 20.7% upon CYC therapy and in 30.0% following RTX within five years after initial diagnosis, yet this difference did not reach statistical significance suggesting that the choice of remission induction therapy did not affect major renal outcomes (Table 3). Similar rates were found for an alternative renal endpoint that is applied in many studies comprising the combination of relapse (at any time), all-cause death and ESKD, with an event rate of 33.7% within five years (CYC 32.4% vs. RTX 42.0%; p = 0.206). Over time, when comparing outcomes between 1999 to 2012 and thereafter, irrespective of the chosen therapies in detail, we observed a decrease in the number of relapses or deaths within 5-year periods (35.8% vs. 27.1%, p = 0.273). However, there was a notable increase in composite renal endpoints (17% vs. 21.6%, p = 0.077, suppl. table 3).

**Table 3:** Influence of therapeutic strategies on relapse rates and occurrence of composite renal endpoints in all cases and AAV subgroups for induction by CYC or RTX or combination

	CYC*	RTX *	RTX + CYC	Other	Total	*p* value*
All cases	*N* = 233	*N* = 60	*N* = 15	*N* = 50	*N* = 358	
Relapse, *n* (%)	83 (35.6)	14 (23.3)	4 (26.7)	23 (46.0)	124 (34.6)	0.071
Relapse or death ≤ 5y, *n* (%)	69 (29.6)	15 (25.0)	3 (20.0)	24 (48.0)	111 (31.0)	0.481
Renal cases	*N* = 188	*N* = 50	*N* = 9	*N* = 17	*N* = 264	
Relapse, *n* (%)	68.0 (36.2)	9.0 (18.0)	1.0 (11.1)	7.0 (41.2)	85.0 (32.2)	0.015
Relapse or death ≤ 5y	57 (30.3)	11 (22.0)	1 (11.1)	7 (41.2)	76 (28.6)	0.247
Composite renal endpoint ≤ 5y	39 (20.7)	15 (30.0)	2 (22.2)	9 (52.9)	65 (24.6)	0.165
Relapse, death or ESKD ≤ 5y	61 (32.4)	21 (42)	4 (44.4)	3 (17.6)	89 (33.7)	0.206

*Pearson ‘s $\chi$ ^2^ test between CYC and RTX

CYC, cyclophosphamide; RTX, rituximab; composite renal endpoint comprises death, demanding dialysis, entering end stage kidney disease (eGFR < 15 ml/min/1,73m^2^) or renal transplantation

**Fig. 3:**
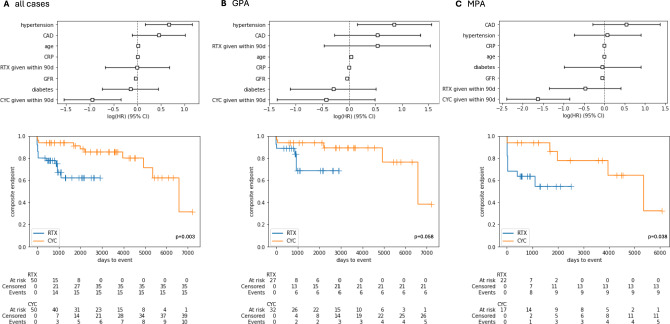
Risk factors for renal events and composite endpoint analyzes in all AAV patients with renal involvement under RTX as compared to propensity matched CYC counterparts in **A** all cases, **B** GPA patients and **C** MPA patients

Risk factors for the composite renal endpoint were examined by Cox proportional hazard analysis (Fig. 3, suppl. table 4) identifying arterial hypertension as the most important factor (HR 1.94, 95%-CI 1.18 to 3.19, p = 0.009). If GPA and MPA were examined separately, a different picture emerged and yielded no distinct risk factor in MPA patients, whereas in GPA patients, arterial hypertension and higher age conferred an elevated risk for adverse renal outcomes (Fig. 3B). Instead, in this entity, remission induction using CYC was associated with a significantly decreased risk to encounter the composite renal endpoint (HR 0.20, 95%-CI 0.09 to 0.43, p < 0.001) in comparison to RTX (HR 0.62, 95%-CI 0.26 to 1.50, p = 0.291, suppl. table 5C).

### Propensity-matched analysis

We were able to confirm a potentially different treatment response by applying Kaplan-Meyer analysis with log-rank testing in all available cases with renal involvement that were induced by RTX (n = 50) in comparison to their propensity-matched CYC-induced counterparts (n = 50) considering age, sex, CRP, BVAS and eGFR at baseline (Fig. 5, suppl. table 2). We observed significantly less patients meeting the renal endpoint upon CYC in total (Fig. 3A, p = 0.003) and in MPA (Fig. 3C, p  = 0.038), but not in the GPA patients (Fig. 3B, p  = 0.058). Among MPA patients who received RTX as induction regimen, a substantial and further decline in renal function occurred shortly after baseline, resulting in an early accumulation of renal endpoint events (Fig. 3C), potentially related to comparably low eGFR values in this group already at baseline (44.5 ml/min/1.73m^2^ in RTX vs. 40.3 ml/min/1.73m^2^ in CYC).

## Discussion

With the present retrospective data analysis from a large, multicentric cohort of AAV patients we thought to shed light on several clinical key aspects and developments obtained from ‘real-world’ observations over more than 20 years. Regarding prior data, major demographic and clinical characteristics, disease expression patterns and comorbidities were similar between our study population and other European AAV cohorts [12–14]. As compared to GPA, the proportion of MPA cases among our entire AAV cohort increased from 29.1% in the early period until 2013 up to 44.8% thereafter. Of note, these percentages do not represent true incidence rates which was beyond the scope of our project. However, they do suggest an increase in the ratio of MPA cases over the last decades. Notably, this is in line with recent observational studies and RCTs in Caucasian participants demonstrating a consistently greater proportion of MPA cases than their GPA counterparts (e.g. in the Mass General Brigham AAV cohort [15] and also in the ADVOCATE [16] cohort recruiting between 2017 and 2018, although GPA was once regarded the predominant AAV variant in Caucasians [8].

Furthermore, in our patients, kidney involvement was more frequent in MPA than in GPA patients and renal function as determined by lower eGFRs at the time of diagnosis by more than 20 ml/min/1.73m^2^. Of note, MPA patients with renal manifestation were more likely to develop unfavorable renal outcomes over a five-year period, particularly in those MPA cases that received RTX as remission induction.

MPA is known to be associated with a higher risk for ESKD as compared to GPA [16] irrespective of age, sex, and baseline renal function [17].This was evident in our cohort revealing an early and comparatively steep decline in renal survival in MPA cases within the first year. Overall, GPA patients experienced significantly more and earlier relapses, whereas the choice of CYC or RTX as remission induction agent did not affect relapse rates. Higher relapse rates for GPA patients had also been observed in prior studies demonstrating a nearly two-fold higher relapse rate in this entity [18, 19]. Prior analyzes from the pre-RTX era showed worse survival and renal outcomes for GPA cases as compared to their MPA counterparts in a large German AAV cohort [20].

In the long-term observation, the randomized RITUXVAS trial analyzed a combined endpoint comprising relapse, death and ESKD occurring within two years after diagnosis and demonstrated no significant difference between CYC and RTX [21]. In our AAV cohort, we analyzed relapse-free survival rates over a distinguishably longer period of five years, which were significantly higher among GPA cases, yet relapse-free survival was not different between patients receiving CYC or RTX in line with data from RITUXVAS. RTX appears to be effective in preventing relapses [22] and nowadays, the use of RTX is clearly recommended as first-line choice for both induction and maintenance therapy by the ACR/EULAR recommendations [23].

A post-hoc analysis of the RAVE trial found a better response in terms of remission upon RTX induction in GPA patients after six months in comparison to CYC/AZA [24]. In the context of relapse prevention, Guillevin et al*.* [22] reported longer relapse-free intervals of more than two years in patients treated with RTX for maintenance following remission induction by CYC as compared to those treated with azathioprine. It seems noteworthy, that most cases were predominantly classified as GPA with a smaller representation of MPA cases. By contrast, our MPA patients generally had a higher renal disease burden and a higher risk to early-on progress towards the composite renal endpoint, particularly when treated with RTX.

Due to the low frequency of only fifteen patients, our analyzes do not allow for a clear evaluation on the combination of RTX and CYC as remission induction strategy. Nonetheless, limited data from prior studies showed that this ensemble might exert additional beneficial effects in preventing renal events and relapses. The combination demands weighing against increased risk of either toxicity or infectious complications. A lower risk of death, development of ESKD or relapse was described by McAdoo et al*.* [3] who investigated this combination in 66 renal AAV cases in a single-center study in comparison to propensity-matched cases of the European Vasculitis Study Group. In terms of infection risk, a recently published study found no difference in the frequency of severe infections between CYC versus RTX but significantly more infections under the combination of both [24]. This needs consideration particularly since MPA patients tend to be older as confirmed in our study and therefore more vulnerable to infectious complications. Another alternative might be the early addition of novel therapeutics such as the C5a complement receptor antagonist avacopan which can now be used as add-on therapy in combination with RTX or CYC in AAV patients.

Secondary analyzes in pre-defined subgroups of the ADVOCATE trial, which led to the license of avacopan in 2022, suggest that avacopan is particularly effective in MPA patients and in relapsing cases [16]. It appears intriguing to evaluate this approach in a prospectively designed RCT specifically in MPA patients. The significant differences suggest that the potential benefits may outweigh putative sample size limitations, particularly when focusing on renal endpoints.

In contrast to our results, Wallace et al*.* found similar rates for kidney failure, death and severe infections between patients treated with RTX or CYC [15] in a large retrospective observational study of 600 AAV patients with almost 70% showing renal involvement and a clear MPO preponderance. Our study includes fewer cases but utilizes follow-up visits in each center instead of registry data for dialysis or death. Propensity matching was applied to patients with biopsy-proven, renal involvement, resulting in small but distinct subgroups. Another contributing factor for this discrepancy may be the relevant number of patients diagnosed as renal-limited AAV cases in clinical diagnoses. These patients account for a significant number of early renal events and are reclassified as MPA according to ACR/EULAR 2022 criteria in almost all cases. Furthermore, our patients exhibited significant variations in maintenance therapies between both cohorts which limit their comparability. However, evaluating the outcomes concerning ANCA type and standardized therapy for both induction and maintenance simultaneously would require a randomized and prospective trial.

The strength of our study relates to its comprehensive analysis of long-term observational data from a large and relatively homogenous European AAV cohort in a 'real-world' context. However, it is important to acknowledge the limitations of the retrospective design. Notably, the number of deaths may have been underestimated due to the absence of death registry data. Data were collected from routine visits, resulting in several censored cases. Nevertheless, patients received close monitoring in large tertiary referral hospitals with core nephrology expertise, which enhances the representativeness of renal events, but on the other hand might constitute a potential selection bias. Moreover, the study was challenged by the heterogeneity and a wide spectrum of combination therapies used in clinical practice. Another limitation of our study relates to a selection bias as we excluded EGPA patients since we aimed to focus on AAV forms with predominant renal affection.

In summary, our analysis advocates for more individualized therapeutic approaches using adjunctive treatment beyond RTX in MPA patients with significant renal involvement. These patients experience more adverse renal events, if treated exclusively with RTX instead of CYC, even though, our data strongly support effectiveness of RTX in induction and preventing relapses in both GPA and MPA patients.

## Supplementary Information

Below is the link to the electronic supplementary material.

Supplementary file1 (DOCX 336 KB)

## Data Availability

All data supporting the findings of this study are available within the paper and its Supplement. Individual patient data are not openly available due to sensitivity reasons but may be available from the corresponding author upon reasonable request.
