# Supplementary material for: Remission induction therapies and long-term outcomes in granulomatosis with polyangiitis and microscopic polyangiitis: real-world data from a European cohort
Source: Rheumatol Int. 2024 Dec 24;45(1):7. doi: 10.1007/s00296-024-05757-4 (PMC11668861; doi:10.1007/s00296-024-05757-4)
Supplement: Supplementary file 1 — Supplementary file1 (DOCX 336 KB) [file 296_2024_5757_MOESM1_ESM.docx]

**Supplemental Material to:**

Krämer *et al.* Remission induction therapies and long-term outcomes in ANCA vasculitis patients: Real-world data from a European cohort

**Suppl. table 1**. Re-classification of AAV patients (comparing initial clinical diagnosis and diagnosis based on the 2022 ACR/EULAR criteria.

| Clinical Diagnosis |  | ACR/EULAR 2022 |  | Concordance  GPA | Concordance  MPA |
| --- | --- | --- | --- | --- | --- |
| GPA | 228 | 208 (-20) |  | 204 |  |
| MPA | 98 | 139 (+41) |  |  | 96 |
| Renal limited Disease | 32 |  |  | MPA: 29 / GPA: 3 |  |
| Not classified | - | 11 (+11) |  |  |  |
| **Total** | 358 | 358 |  |  |  |

**Concordance relates to cases subgrouped to the same disease entity in the initial clinical diagnosis and following the ACR/EULAR 2022 criteria.**

**
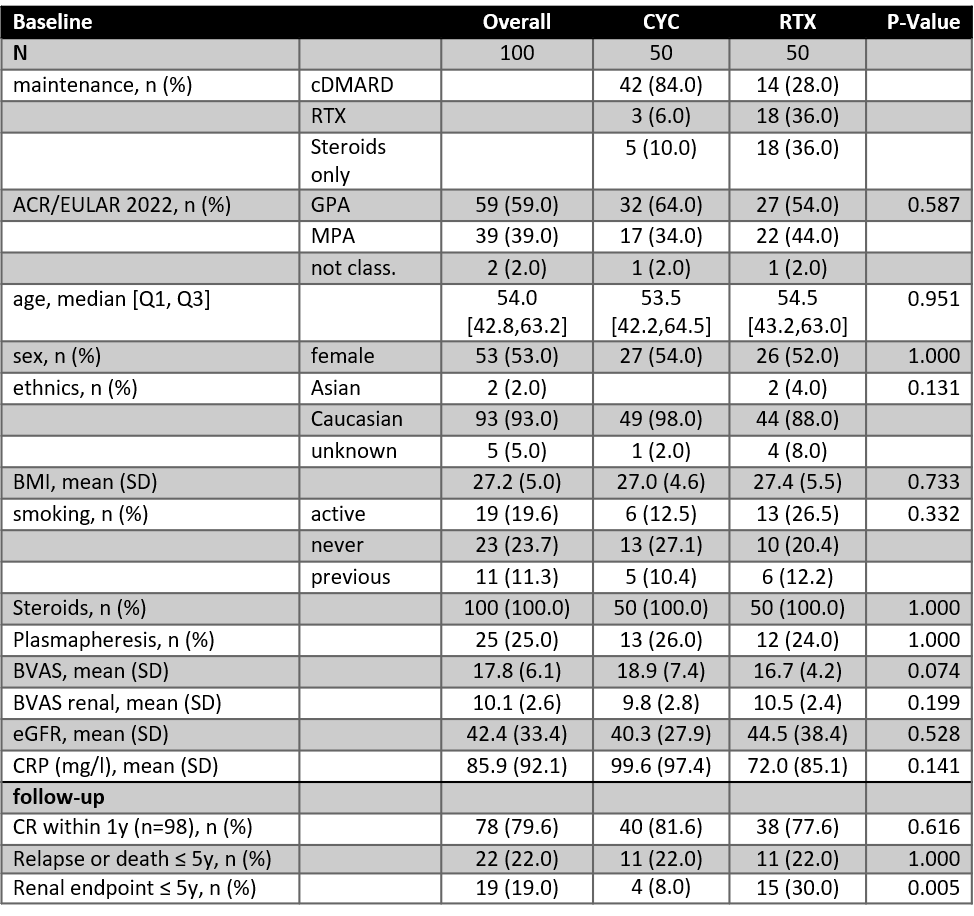
**

*eGFR, stimated glomerular filtration rate (ml/min/1,73m²)*

*cDMARD, conventional disease modifying dugs for maintenance after CYC: AZA 28 (56%), MMF 11 (22%), MTX 2 (4%), Leflunomide 1 (2%) and after RTX: AZA 7 (14%), MMF 3 (6%), MTX 2 (4%) , Leflunomide 2 (4%).*

**Suppl. table 2**. Baseline characteristics and outcome of RTX-treated renal AAV cases compared to propensity matched CYC-cases.

**
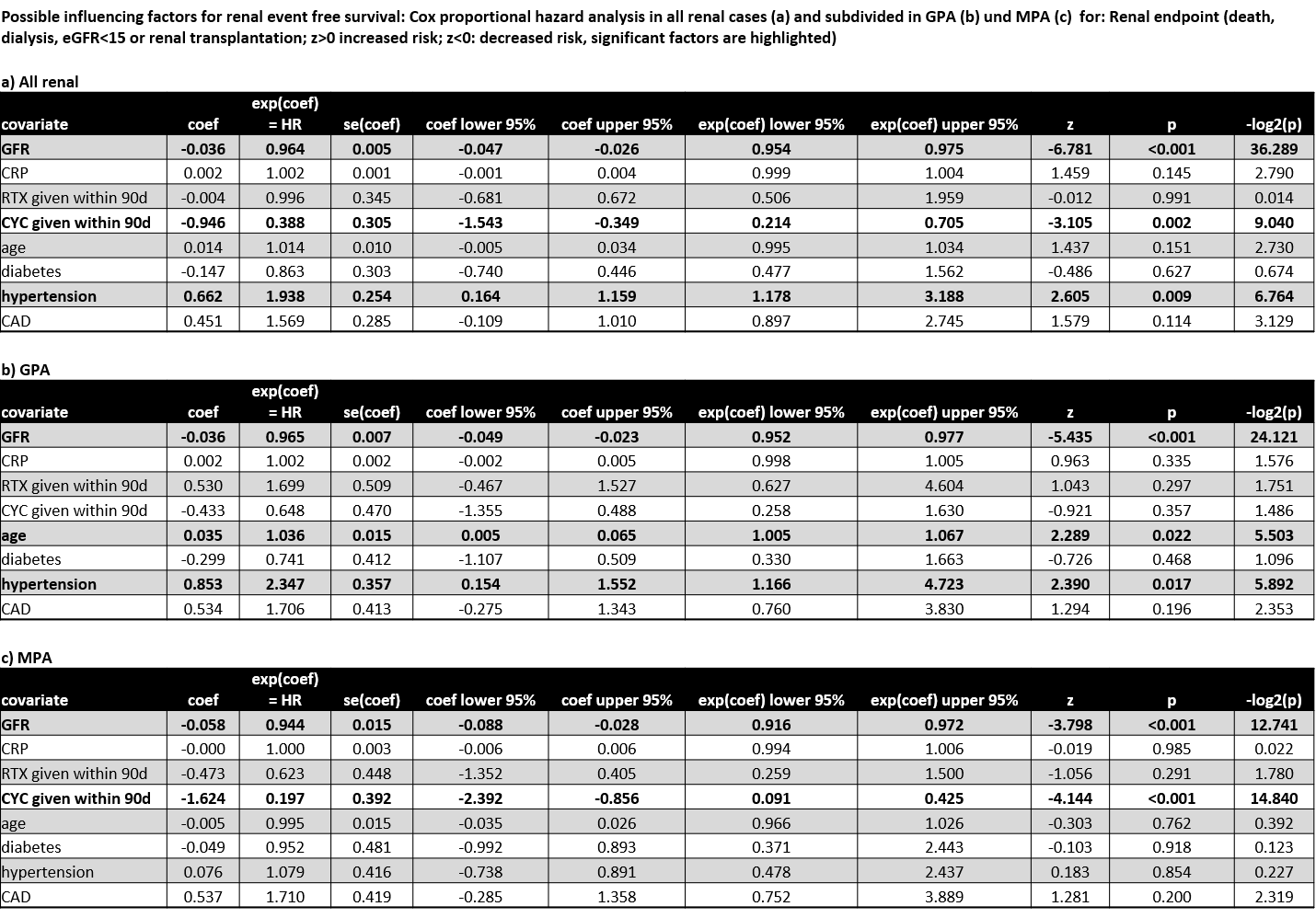

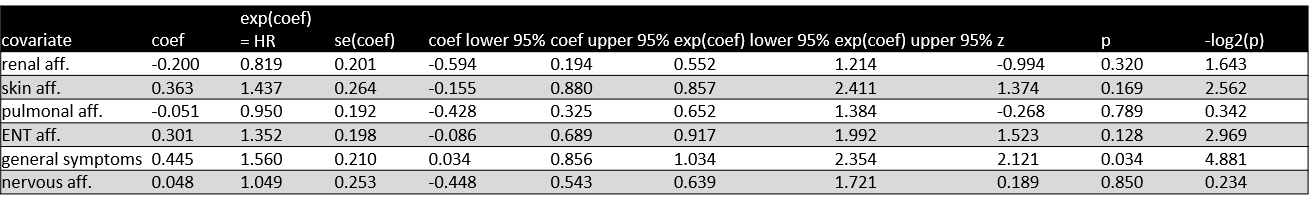

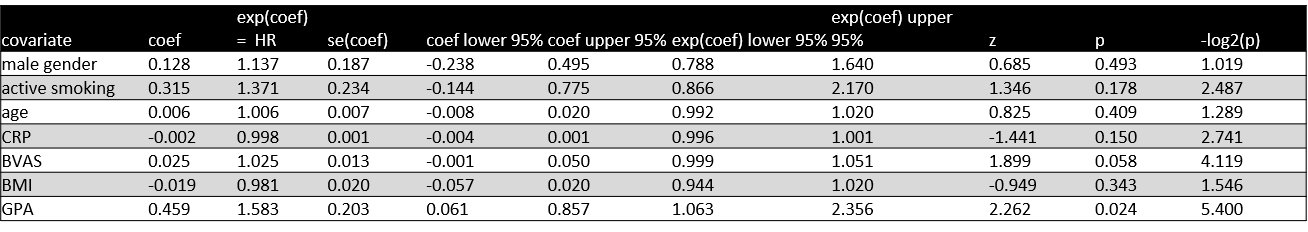
**

**Suppl. table 3.** Therapeutic regimen before and after 2013.

**Suppl. table 5.** Possible influence factors for renal event-free survival

**Suppl. table 4.** Risk factors for disease relapse.
